# Ayurveda in Argentina and other Latin American countries

**DOI:** 10.4103/0975-9476.72614

**Published:** 2010

**Authors:** Jorge Luis Berra, Rosana Molho

**Affiliations:** 1 *Fundación de Salud Ayurveda Prema, Buenos Aires, Argentina*; 2 *School of Medicine, University of Buenos Aires and National University of Cordoba, Argentina*

**Keywords:** Ayurveda, Argentina, Latin America, education, local medicinal plants

## Abstract

Over the past 20 years the Fundacion Salud de Ayurved Prema Argentina has spread the knowledge of Ayurveda throughout Latin America. The Fundacion is based in Buenos Aires in the Argentine Republic, where it now runs courses in two of the country’s major medical schools - at the School of Medicine of the University of Buenos Aires, and the National University of Cordoba’s School of Medicine. Based on an MoU with Gujarat Ayurveda University, at Jamnagar, Gujarat, the Fundacion has been accredited as a Collaborating Center for teaching, assistance and research in the field of Ayurvedic Medicine in Argentina. This has led to successful missions to other countries in the region where the Fundacion and its associates have been able to start dialogues with governments, and in places hold sizeable courses. The knowledge of Ayurveda is now spreading throughout South and Central America and hardly a country remains untouched by it.

## INTRODUCTION

The Fundación de Salud Ayurveda Prema (Prema Ayurveda Health Foundation) is a nonprofit organization registered with the Argentine government. Its goal is to contribute to the promotion of Ayurveda medicine and all its preventive and therapeutic resources of value in the health field. The aim of the Foundation is to help Ayurveda attain the best possible status within Argentina’s medical community and with the general public. Our vision is to improve and perfect the health of the peoples of Latin America using Ayurveda.

We have now been spreading Ayurveda in the Argentine Republic and Latin America for over 20 years, focusing our efforts on both the medical profession and the people. Throughout this time, our interest has been to integrate useful elements of traditional approaches to medicine such as Ayurveda, which is our specific field of knowledge, with mainstream medicine and scientific research methods in an atmosphere of mutual respect, acknowledgment, and positive assessment. Currently, we are conducting four postgraduate courses on Ayurvedic medicine, all certified by the most prestigious universities in the country. In the fields of teaching and research, our goal is to preserve the wealth and purity of Ayurveda’s teachings, and to demonstrate its usefulness for modern biomedical science.

## COLLABORATION WITH GUJARAT AYURVEDA UNIVERSITY

On January 5, 2001, after obtaining approval from India’s Ministry of Foreign Affairs and Ministry of Health, the Fundación de Salud Ayurveda Prema signed a Memorandum of Understanding (MoU) with Gujarat Ayurveda University (Jamnagar, India) in the presence of Shailaja Chandra (Secretary, Indian System of Medicine and Homeopathy), the Governor of the Gujarat State, and other top Indian officials. The MoU accredited the foundation as a collaborating center for teaching, assistance, and research in the field of Ayurvedic Medicine in Argentina.

In 2000, the School of Medicine of the University of Buenos Aires approved our first postgraduate course on Ayurvedic medicine.[1] Founded in 1821, the University is the most important higher education institution in Argentina. Its 13 schools or departments and 6 affiliated hospitals comprise around 312,000 students and 30,000 professors and lecturers in 108 fields. Among its professors and scholars there have been five Nobel Prize winners. On October 18, 2000, Jorge Luis Berra, Director of the Course, gave a lecture about “Ayurveda in the XXI century” at the opening ceremony, with the participation of Nigan Prakash, Indian Ambassador in Argentina. Mrs. Shailaja Chandra, then Secretary of the Department of the Indian Systems of Medicine and Homeopathy, Ministry of Health and Family Welfare, Government of India, informed us that, as far as she knew, this was the first postgraduate course on Ayurveda given at a public university in a Western country.

Officials from five different departments of the School of Medicine had examined the course program for a year. Without exception, they deemed it of interest to the medical community as a whole. They considered that it had appropriate objectives, a well-developed analytical curriculum, sufficient instructional resources, an adequate schedule, as well as good infrastructure and assessment procedures. The course was finally approved by the School Board and the Dean. It is intended for medical doctors, psychologists, pharmacists, and other professionals in the health field. Several Indian ambassadors to Argentina like Pramathesh Rath (2006 and 2007) and R. Viswanathan (2008) have also delivered lectures to the course.

Later, in 2002, an annual advanced postgraduate course on Ayurvedic medicine, accredited by the University’s School of Medicine and designed for health professionals, was added.[2] That same year, another resolution[3] from the Board of Directors of their School of Medicine approved a postgraduate distance learning course on Ayurvedic medicine. All these courses have been delivered under the auspices of the Indian Embassy in Argentina, and, since 2005, with the authorization of the then Indian Ambassador, Ringzind Wangdi.

Well-known and respected Ayurvedic scholars and doctors from leading Indian institutions have supported the courses through their articles, including, among others, former Vice-Chancellor, Rajasthan Ayurveda University, R. H. Singh; former Vice-Chancellor, Gujarat State Ayurved University, and Ayurvedic Advisor, Dept. of *AYUSH*, S. S. Savrikar; Director of the Institute of Postgraduate Teaching and Research, and former Vice-Chancellor, Gujarat State Ayurved University, M.S. Baghel; Director National Institute of Ayurved, Jaipur, Ajay Kumar Sharma; and Professor of Ayurveda, Gujarat State Ayurved University, H. M. Chandola.

Support of top academics from India’s major educational institutions devoted to Ayurvedic studies has made it possible for us to transmit Ayurveda’s fundamentals and its most relevant therapeutic tools completely faithfully. It has also enabled us to enrich course content through scientific interchange with well-known institutions at the highest academic level. All this was made possible by the MoU with Gujarat State Ayurveda University.

## RECENT INITIATIVES

In April 2010, a new biennial postgraduate course on Ayurvedic medicine exclusively for medical doctors was started at the National University of Cordoba’s School of Medicine.[4] The University, founded in 1613, is the oldest in Argentina, and one of the first in the Americas. Its academic structure includes 12 schools, 98 research institutes, 20 libraries, 14 museums, and 2 astronomical observatories. Its faculty comprises 8203 professors and lecturers; 104,218 students are enrolled in its 90 undergraduate courses.

More than 450 professionals, 250 of whom were medical doctors, have now taken our postgraduate courses, while another 500 nonprofessional students have participated in courses on Ayurvedic principles and Ayurvedic massage. Students come from many countries in addition to Argentina, including Uruguay, Brazil, Paraguay, Chile, Bolivia, Peru, Ecuador, Venezuela, Colombia, Panama, Costa Rica, Mexico, Spain, Portugal, Italy, and the USA.

For the last 3 years, we have been participating in an optional Course on Complementary Medicine for students of the School of Medicine at the University of El Salvador, in Buenos Aires. We also run regular courses on Ayurvedic massage for health technicians. These give technicians a more integrated and holistic approach to patient care, and have attracted great interest.

Since 2005, we have also offered online courses on the principles of nutrition in Ayurveda. By implementing several educational strategies that establish a more personal relation with students, and so replacing the direct contact in normal courses, we have achieved a very positive response. This has enabled our students, who come from all Spanish-speaking countries, to share their personal growth and transformation experiences; the latter often extend to their families, making it possible for whole family groups to acquire better health habits.

We have also conducted lectures, seminars, workshops, and conferences on various Ayurveda topics in Ministries of Health, Schools of Medicine, and professional and scientific associations in most Latin American countries: Mexico, El Salvador, Honduras, Nicaragua, Costa Rica, Panama, Colombia, Venezuela, Ecuador, Peru, Brazil, Paraguay, and Chile, even Spain and India [Table 1].

**Table 1:** Activities carried out in different countries

Year	Country	Activity
2001	India	MoU with Gujarat Ayurved University (1/2001).
		Speaker at IInd. Intl. Seminar on Ayurveda (1/2001) and at the National Seminar on Ayurveda and Mental Health (7/2001),
		Gujarat Ayurved University
2002	Panama	“Integrating Alternative and Allopathic Medicine” and “Scientific Basis of Phytotherapeutics Treatments” (Key Note Speak);
		“Dietetics and Ayurveda” (Workshop). II° Symposium of Naturopathy organized by the Panamanian Tourism Institute
		(1/2002).
		“Contributions of the Ayurveda to the health in the XXI Century.” Organized by the Panamanian Institute of Tourism,
		Panamanian Government. “Introduction to the Ayurveda” Universidad de las Americas. (7/2002)
		“Ayurveda and Nutrition” in the Diplomate of Alternative Therapies, Health and Tourism organized by the Universidad de
		las Americas and some governmental agencies. “Introduction to Ayurveda” (11/2002)
2002	Costa Rica	Conference “the Ayurvedic Drugs” destined to the Commission of Natural Products and professionals of the Ministry of
		Health of Costa Rica.
		“Contributions of the Ayurveda to the health in the XXI Century.” University of Costa Rica.
		“Contributions of the Ayurveda to the health in the XXI Century.” Latin University of Costa Rica.
		“Therapeutic uses of the Ayurvedic medicines.” Seminar and Workshop
2003	India	“Ayurveda in Latin America” International speaker at Ayurveda World Summit (Bangalore).
		“International Acceptance of Ayurvedic Remedies.” International speaker at the Congress Recent Advances in Ayurveda,
		Faculty of Ayurveda, IMS, Banaras Hindu University
2003	Brazil	“Ayurveda and Antioxidants,” International Symposium of Orthomolecular Medicine, organized by the Brazilian
		Orthomolecular Society, (10/ 2003)
2003	Paraguay	Lectures at different Institutions (6,9 and 11/2003)
2003	Peru	Lectures at different Institutions (7,9/2003)
2004	India	Coordination of the Intensive Training Seminar on Ayurveda for Argentinean and Spanish speaking medical doctors with
		professors of IPGTR, Gujarat Ayurved University
2004	Costa Rica	“Ayurvedic Phytotherapy” at the first Course of Clinical Phytotherapy, organized by the Faculty of Medicine, Costa Rica
		University in association with the National Medical Board
		“Uses of Ayurvedic Phytotherapy,” Faculty of Health Sciences, School of Medicine, Universidad Internacional de las
		Americas
		“Mechanism of action of Ayurvedic Remedies,” Ministry of Health, Drug Registration Commission
		“Uses of Ayurvedic Phytotherapy,” Faculty of Medicine, Autonomous University of Central America
		“Uses of Ayurvedic Phytotherapy,” Faculty of Medicine, Universidad Latina
		“Phytochemical basis of Ayurvedic Remedies,” National Board of Pharmacists
		“Contributions of Ayurveda at the XXI century,” Universidad Internacional de las Americas
2004	Honduras	“Introduction to the Ayurvedic Phytotherapy,” Meeting at the Faculty of Pharmacy, Universidad Autonoma de Honduras
		“Scientific Basis and Mechanism of Actions of Herbomineral Remedies,” National Board of Pharmacists
2004	Nicaragua	“Scientific Basis and Mechanism of Actions of Ayurvedic Remedies,” Faculty of Pharmacy, Autonomous University of
		Nicaragua.
		“Phytochemical basis of Ayurvedic Remedies,” Health Ministry
2004	Venezuela	“Scientific Basis and Mechanism of Actions of Ayurvedic Remedies.” Workshop carried out at the Ministry of Health for the
		professionals of the Health Department, Public Health Department and the Registry Commission of Natural Products
2004	Peru	“The science of Ayurveda.” Centro AyurVeda
2005	India	Coordination of the Intensive Training Seminar on Ayurveda for Argentinean and Spanish speaking medical doctors with
		professors of IPGTR, Gujarat Ayurved University
2005	Brazil	“The science of Ayurveda.” Facultade Espirita
2005	Chile	“Ayurvedic Medicine.” Collaborating Professor, Course on Complementary Medicine, Faculty of Medicine, University of Chile
2006	India	Coordination of the Intensive Training Seminar on Ayurveda for Argentinean and Spanish speaking medical doctors with
		professors of IPGTR, Gujarat Ayurved University.
		International Speaker at the V International Seminar organized by Gujarat Ayurved University
		“Physiology of Meditation.” International Speaker at the International Conference on Ayurveda, Mahabbhalipuran,
2006	Chile	“Ayurvedic Medicine.” Collaborating Professor, Course on Complementary Medicine, Faculty of Medicine, University of Chile
2007	India	Coordination of the Intensive Training Seminar on Ayurveda for Argentinean and Spanish speaking medical doctors with
		professors of IPGTR, Gujarat Ayurved University
2007	Chile	“Ayurvedic Medicine.” Collaborating Professor, Course on Complementary Medicine, Faculty of Medicine, University of Chile Annual Course on Ayurveda. Centro Narayan
2007	Ecuador	Ayurveda in the XXI Century.“ Universidad Andina, Quito Intensive Seminar of Ayurvedic Medicine. Hospital de Los Valles, Quito
2007-2008	Colombia	Intensive Seminar of Ayurvedic Medicine. Narayan Centre (10/2007 to 2/2008)
2008	Spain	Ayurvedic Massage Course. Madrid
2008	Brazil	”Physiology of Meditation.“ International Speaker at the II Congreso Brasileiro de Ayurveda organized by Asociacion Brasileira de Ayurveda. San Pablo (7/2008) ”Ayurvedic Treatment of Diabetes.“ International Speaker at the 1st International Congress ofYoga and Ayurveda. San Pablo (8/2008)
2008	Chile	Annual Course on Ayurveda. Centro Narayan
2009	Chile	Annual Course on Ayurveda. Centro Narayan
2009	Ecuador	Intensive Semminar of Ayurvedic Nutrition, Casa de la Cultura Ecuatoriana Intensive Course on Ayurvedic Massage, Satori Centre
2009	Mexico	Intensive International Seminar on Ayurveda, National Medical Center
2010	Chile	Annual Course on Ayurveda. Centro Ayurveda

For example, in 2002 we coordinated and taught a course at the University of the Americas, Panama. The course, which led to a “Diploma in the Ayurveda Nutrition System, Manual Therapies and Body Aesthetics for Healthier Tourism,” was conducted with assistance from the Panama Institute for Tourism. We also conducted training workshops for low-income families on the Ayurvedic use of local medicinal plants. Another example: in 2006 and 2007, we were invited to take charge of Ayurveda training at an Online Course on Complementary Medicine at the School of Medicine, National University of Chile.

## OFFICIAL RECOGNITION BY GOVERNMENTS

A crucial aspect of successfully introducing Ayurveda practice in Latin America has been our approach to Ayurvedic medicines. We have counseled the appropriate Health Departments within the Health Ministries of Venezuela and Costa Rica to give them scientific information, and help their personnel understand the background of the therapeutic use of such medicines, thus facilitating their official registration.

At present, Ayurvedic medicines produced in India are officially registered in Venezuela, Costa Rica, Nicaragua, and Honduras. In other Latin American countries, we make use of local herbal medicines. We have taken immense trouble to make accurate correspondences between local medicinal plants and those originating in India. For example, we have developed a list of medicinal plants from Latin America and the West that can be used in Panchakarma procedures.[5]

## WESTERN MEDICINAL PLANTS FOR PANCHAKARMA [TABLES 2–8]

**Table 2:** Medicinal plants for Snehana

Botanical name	Spanish name	Ayurvedic name
*Brassica oleracea*	Berza	Daladamini
*Malva sylvestris*	Malva	Khubaji

**Table 3:** Medicinal plants for Swedana

Botanical name	Spanish name	Ayurvedic name
*Carthamus tinctorious*	Cartamo	Kusumbha
*Ruta graveolens*	Ruda	Sitaba

**Table 4:** Medicinal plants for Vamana

Botanical name	Spanish name	Ayurvedic name
*Acorus calamus*	Calamo aromatico	Vacha
*Viola odoranta*	Violeta	Vanafsha (Root)

**Table 5:** Medicinal plants for Virechana

Botanical name	Spanish name	Ayurvedic name
*Ipomoea purga*	Jalapa	Jaalapaa − Julafa harada
*Taraxacum officinale*	Diente de leon	Dugdhafeni

**Table 6:** Medicinal plants for Anuvasana Vasti

Botanical name	Spanish name	Ayurvedic name
*Foeniculum vulgare*	Hinojo	Mishreya
*Hordeum vulgare*	Cebada	Yava

**Table 7:** Medicinal plants for Aasthapana Vasti

Botanical name	Spanish name	Ayurvedic name
*Alpinia officinarum*	Galanga	Kulanjana − Rasna variety
*Anethum graveolens*	Eneldo	Shatapushpa

**Table 8:** Medicinal plants for Nasya Karma

Botanical name	Spanish name	Ayurvedic name
*Acorus calamus*	Calamo aromatico	Vacha
*Origanum majorana*	Mejorana	Marubaka

In order to spread Ayurveda most effectively, we took a step we regarded as essential: translation of some of the important, classical Ayurveda texts into Spanish, the second most spoken language in the world. Similarly, we brought out Spanish language publications on distance courses on Ayurvedic medicine,[6–9] and a book[10] for the general public on the philosophical and practical basis of Ayurveda. The Sociedad Argentina de Pediatría (Argentine Society for Pediatrics) asked us to collaborate with it in its CME Program on Pediatrics, attended by more than 60% of its members. Twelve topics for professionals’ permanent training are selected each year. In 2009, the choice fell to Ayurveda, together with some other complementary medical systems.[11]

The translation of the six volumes of Charaka Samhita,[12] a task that has taken several years, is in its final revision stage and will soon be published. Translation of Susruta Samhita has already begun. In this way, the Fundación de Salud Ayurveda Prema is making a modest but relevant contribution to correct knowledge of Ayurveda in this region of the world. We have also assembled a collection of the fundamental texts of Ayurveda in English. More than 500 classical and modern Ayurveda texts translated into English have already been indexed.

Some papers resulting from our research work and surveys on different subjects, such as “The International Acceptance of Ayurvedic Formulations,”[13] “Physiology of Meditation,”[14] “Anorexia Nervosa and Ayurveda,”[15] and “Attitudes of Health Professionals Towards Non-conventional Medicine: An Argentinean Survey,”[16] have been presented in Indian and Latin American Congresses and Conferences. At the same time, medicinal plants from Latin America and the West are the subject of ongoing studies, in which herbal medicine concepts and phytomedicine research are integrated with Ayurvedic knowledge.

Our relationship with Gujarat Ayurveda University has led to four Intensive Training Seminars in Jamnagar, in which students from our own Foundation, other Latin American countries, and Spain have participated. The Seminars included training classes, observation of Ayurvedic procedures, visits to botanical gardens, and scientific exchanges with professors of the Institute of Post Graduate Teaching and Research in Ayurveda, as well as with other teachers at the University. Reciprocally, Indian professors and experts from many Western countries, such as Subhash Ranade and Robert Svoboda, have participated in workshops and seminars in Argentina.

As a result of our postgraduate courses, and seminars conducted in many countries of the Latin American region, and in India, some of our students have been able to establish new Ayurvedic centers in their countries of origin.

## AYURVEDA: A TIMELY SOLUTION TO MODERN HEALTHCARE CHALLENGES

Ayurveda offers practical solutions to many problems in modern life. In Argentina, as in other countries in the last few years,[17,18] there has been increasing interest and acceptance of our work everywhere. Among the challenges of the new millennium are treatment of chronic illnesses, and new ways of fostering personal health and well-being. Ayurveda, through its emphasis on the whole person, and its proposal of a reasonable and rational lifestyle giving attention to nutrition, daily routines, exercise, and mental harmony, offers useful resources to professionals wishing to take advantage of its millennia-old wisdom. Besides, its pharmacology based on natural products offers an effective and well-tolerated means to managing certain diseases.

Put to the test in several cultures, Ayurvedic principles have proved useful in both prevention and cure the world around. Our course participants come from many countries, and have subsequently attested to the validity and wisdom of these principles.

The World Health Organization has recognized Ayurveda to be a very sophisticated system of traditional medicine.[19] Ayurveda is a study of life that stimulates observation, and fosters scientific research. Conventional studies have corroborated many of its ancient postulates.[20] Currently, the interest of both physicians and patients is increasing in traditional systems of medicine.[21,22] All of them need to count on knowledgeable professionals, well-trained in new kinds of care, and able to provide answers to humanity’s modern health problems. Demand for training and information is high.

What the new millennium specifically requires is integrated training of health professionals to fulfill increasing demands in the West. Health professional teams, patients, and the general public are all experiencing a deep need for a system that integrates body, mind, and spirit. Our time demands a personalized approach to prevention, and effective, natural methods of treatment, with low levels of side effects or adverse responses.

In the world’s most prominent universities, there is currently a trend to include courses on complementary and alternative medicine. A 1998 survey of American schools of medicine found that, of the 117 which answered the questionnaire out of 125 surveyed, a total of 123 courses were taught at 64% of the university medical schools.[23]

It has always been held that universities are the proper place for evolution of thinking as they offer an environment of freedom for discussion of ideas, and encouraging the advancement of science. Our postgraduate courses have been approved since they offer a framework for mutual respect and tolerance for different ways of viewing health and treating illness. As narrated above, these considerations have led to our courses being recognized by Argentina’s best traditional learning institutions such as the University of Buenos Aires and the National University of Cordoba.

The World Health Organization has urged that interchanges between traditional and modern medicine be promoted.[24] To achieve this goal, members of health teams need tested information sources and methods. Without compromising professional responsibility or scientific principles, some collaborative projects have begun to take form. This joint work will make it possible to distinguish therapies useful for patient care from others. In addition, it will require developing special abilities, valuing sources of knowledge focused on ways to maintain and preserve health.

Of all systems of traditional medicine, Ayurveda seems to be best understood by people in Latin America and the West, and thus most up-to-date. Experience confirms that its ancient wisdom is easily accepted and incorporated both by professionals, health workers, and the general public. We find increasing numbers of people and institutions turning to its concepts and resources to care for individuals and families, and to heal disease. Participants in our courses tell us that Ayurveda’s holistic and scientific approach improves and deepens the practical work of physicians, psychologists, nutritionists, physiotherapists, kinesiologists, nurses, yoga teachers, nutrition counselors, manual therapists, and other health professionals and technicians.

Fundación de Salud Ayurveda Prema is open to possible forms of collaboration with other Ayurvedic institutions: (a) in order to organize training visits for its members to Ayurvedic centers in India, and visits of foreign Ayurvedic professors to Argentina; (b) to carry out scientific research projects to demonstrate to the biomedical community the value and usefulness of Ayurvedic resources and remedies; and (c) to support work on the provision of Ayurvedic texts in Spanish.

Ayurveda has shown its potential to solve some of our age’s greatest health problems. We are glad to be a part of this movement. Those trained in this ancient Science of Life will be at the forefront of a highly positive and enriching trend in the field of health.
